# Clinically informed intermediate reasoning enables generalizable prostate cancer prognostication through machine learning in limited settings

**DOI:** 10.1038/s41746-025-02193-x

**Published:** 2025-12-03

**Authors:** Jun Akatsuka, Kotaro Tsutsumi, Mami Takadate, Yasushi Numata, Hiromu Morikawa, Atsushi Marugame, Hayato Takeda, Yuki Endo, Yuka Toyama, Takayuki Takahashi, Kaori Ono, Junya Iwazaki, Ryuji Ohashi, Akira Shimizu, Tomoharu Kiyuna, Maki Ogura, Masao Ueki, Takuma Kato, Toshiyuki China, Mikio Sugimoto, Hisamitsu Ide, Naoto Sassa, Naonori Ueda, Shigeo Horie, Toyonori Tsuzuki, Go Kimura, Yukihiro Kondo, Yoichiro Yamamoto

**Affiliations:** 1 Pathology Informatics Team, RIKEN Center for Advanced Intelligence Project, Tokyo, Japan; 2 Department of Urology, Nippon Medical School, Tokyo, Japan; 3 Department of Neurology, University of California, Irvine, Orange, CA USA; 4 Mathematical Intelligence for Medicine, Graduate School of Medicine, Tohoku University, Miyagi, Japan; 5 Department of Integrated Diagnostic Pathology, Nippon Medical School, Tokyo, Japan; 6 Department of Analytic Human Pathology, Nippon Medical School, Tokyo, Japan; 7 Medical Solutions Department, NEC Corporation, Tokyo, Japan; 8 School of Information and Data Sciences, Nagasaki University, Nagasaki, Japan; 9 Department of Urology, Faculty of Medicine, Kagawa University, Kagawa, Japan; 10 Department of Urology, Juntendo University Graduate School of Medicine, Tokyo, Japan; 11 Department of Innovative Longevity, Juntendo University Graduate School of Medicine, Tokyo, Japan; 12 Department of Urology, Aichi Medical University Hospital, Aichi, Japan; 13 Goal-Oriented Technology Research Group, RIKEN Center for Advanced Intelligence Project, Tokyo, Japan; 14 Department of Surgical Pathology, Aichi Medical University Hospital, Aichi, Japan

**Keywords:** Cancer, Medical research, Mathematics and computing

## Abstract

Machine learning has shown promise in medical image classification. However, its generalizability remains challenging. Here, we show that data-efficient pre-surgical prognostication of prostate cancer from biopsy specimens is enabled by versatile feature extraction from whole-mount histopathology and a clinically informed intermediate reasoning step. With data from multiple institutions, our pipeline resolved dual-domain shifts across specimen types and institutions and achieved consistent external validation, reinforced by comprehensive analyses of generalizability. This highlights the robustness of our prognostic approach when compared to the Gleason grading system. We establish an equitable, interpretable, and clinically applicable framework, supporting actionable decisions for prognosis and treatment planning, even in limited real-world clinical environments.

## Introduction

Over the last decade, machine learning (ML) algorithms have been successfully applied to medical image classification, enabling the identification of disease patterns^1–4^. For instance, algorithms developed as part of the prostate cancer grade assessment (PANDA) challenge have successfully detected tumors on prostate biopsy histopathology images at a level comparable to that of uropathologists^5^. Foundation models have recently been gaining popularity, including their application to histopathology images^6,7^. There has been a growing movement toward creating extensive datasets for model pre-training and maximizing performance. The evolution of foundation models, based on the transformer architecture that was introduced in 2017, can be observed as reflecting a “scaling is all you need” approach^8,9^.

Previously, we developed an ML algorithm that employed an autoencoder and clustering to extract explainable features from unannotated prostate whole-mount histopathology images in an unsupervised manner^10^. This can be considered an early pioneering framework toward foundation models applicable to medical image analysis. However, inherent limitations remain in acquiring large-scale, meaningful data in the medical informatics field owing to institutional variations in format and acquisition methods. While collecting large amounts of data from a single institution may help address data scarcity, it often does not resolve the challenge of external applicability. This issue is compounded by the heterogeneous nature of medical samples and concerns regarding the privacy and security of healthcare data. Therefore, it is imperative to develop equitable ML systems that maintain interpretability and generalizability across institutions with limited training data availability.

Prostate cancer is the most common malignancy in men worldwide^11^. Radical prostatectomy (RP) is the primary treatment for localized prostate cancer, offering excellent oncologic control; however, approximately 20-40% of patients develop biochemical recurrence (BCR), characterized by an increase in serum prostate-specific antigen (PSA) level^12–14^. Therefore, early and objective prediction of BCR is crucial for both patients and clinicians. The Gleason grading system was introduced in 1966 by Donald F. Gleason and his colleagues at the Minneapolis Veterans Administration Hospital^15^. The system assigns scores from 1 to 5 based on histological patterns of prostate cancer, with the final Gleason score comprising the sum of the two most prevalent patterns. Although the theoretical range spans 2–10, scores below 5 are rarely encountered in clinical practice. The Gleason grading system is widely used as a primary histological evaluation tool to assess prostate cancer. The robustness of the system to domain shifts stems from its ability to standardize prognostication across diverse sample preparations, evaluators, and clinical contexts, thereby facilitating effective risk communication among clinicians in different practice settings. Thus, the fundamental value of the Gleason grading system lies in both its prognostic capacity and standardization of the prognostic process.

This study aimed to implement an intermediate reasoning step in ML models for enhanced and generalizable prognostication of BCR following RP. Our approach is particularly notable for addressing dual domain shifts across both specimen types and institutional sources in limited data settings: feature extraction was based on post-surgical whole-mount sections, while prognostication was conducted using pre-surgical biopsy samples and externally validated on datasets from multiple institutions. Given the scarcity of high-quality annotated data across healthcare institutions, particularly in underserved regions, our approach aims to improve generalizability, interpretability, and clinical applicability, and allow for democratized access to robust ML systems, thereby contributing to digital health equity.

## Results

### Clinical characteristics

A total of 380 patients were enrolled in the study: 270 from Nippon Medical School Hospital (NMSH), 71 from Aichi Medical University Hospital (AMUH), and 39 from Juntendo University Hospital (JUH). Notably, 100 NMSH patients who provided whole-mount sections used for feature generation were excluded from clinical characterization because they were not used for biopsy model validation purposes. The remaining 170 patients with NMSH who provided biopsy samples were utilized for subsequent training and validation. Table 1 displays clinical characteristics and pre-operative pathological variables of patients for BCR predictions. In the NMSH cohort, PSA level (*p* < 0.001) and density (p < 0.001), clinical (*p* = 0.030) and pathological T stages (*p* < 0.001), and pre- (*p* = 0.016) and post-operative Gleason grading (*p* < 0.001) were higher in the BCR group, whereas no significant differences were observed in age, total prostate volume (TPV), and pathological N stage. In the AMUH cohort, PSA density (*p* = 0.044) and pathological T stage (*p* < 0.001) were higher in the BCR group, whereas no differences were observed in age, PSA level, TPV, clinical T stage, pathological N stage, and pre- and post-operative Gleason grading. In the JUH cohort, patients with BCR were older (*p* = 0.011) and had higher clinical (*p* = 0.003) and pathological T stages (*p* = 0.017), with no significant differences in PSA level, TPV, PSA density, pathological N stage, and pre- and post-operative Gleason grading. At NMSH, the biopsy positive core rate was significantly higher in patients who developed BCR than in those who did not (*p* = 0.025), and a similar difference was observed at JUH (*p* = 0.002). The presence of intraductal carcinoma of the prostate (IDC-P) in biopsy specimens was significantly associated with BCR at NMSH (*p* < 0.001), with comparable but nonsignificant trends at AMUH and JUH. The cribriform pattern was also correlated with BCR at NMSH (*p* = 0.003) and AMUH (*p* = 0.033).

**Table 1:** Clinical characteristics of patients for BCR predictions

		NMSH				AMUH				JUH			
		Total	BCR	Non-BCR	*p* value	Total	BCR	Non-BCR	*p* value	Total	BCR	Non-BCR	*p* value
Cases, *n*		170	82	88	–	71	18	53	–	39	24	15	–
Age, year	Mean (SD)	66.7 ± 6.46	66.2 ± 6.58	67.1 ± 6.37	0.38	68.0 ± 6.32	69.6 ± 3.63	67.4 ± 6.95	0.10	66.7 ± 6.00	68.4 ± 6.76	64.1 ± 3.24	0.011
PSA, ng/ml	Mean (SD)	10.9 ± 7.28	14.0 ± 8.33	8.0 ± 4.54	<0.001	9.4 ± 6.24	11.9 ± 7.02	8.5 ± 5.78	0.075	7.8 ± 4.03	8.5 ± 4.55	6.5 ± 2.70	0.088
TPV, cm^3^	Mean (SD)	29.1 ± 13.6	29.0 ± 15.0	29.1 ± 12.1	0.98	36.4 ± 20.3	32.5 ± 12.4	37.7 ± 22.4	0.23	32.1 ± 19.5	31.0 ± 11.6	33.8 ± 28.3	0.72
PSA density, ng/ml/cm^3^	Mean (SD)	0.435 ± 0.350	0.555 ± 0.377	0.322 ± 0.281	<0.001	0.286 ± 0.190	0.383 ± 0.240	0.254 ± 0.159	0.044	0.295 ± 0.195	0.304 ± 0.204	0.281 ± 0.185	0.72
Clinical	T1	51	18	33	0.030	27	7	20	1.000	10	2	8	0.003
T stage	T2≤	119	64	55		44	11	33		29	22	7	
Pathological	T2≥	74	24	50	<0.001	47	5	42	<0.001	24	11	13	0.017
T stage	T3≤	96	58	38		24	13	11		15	13	2	
Pathological	N0	166	79	87	0.35	69	16	53	0.062	37	22	15	0.51
N stage	N1	4	3	1		2	2	0		2	2	0	
Pre-operative	7≥	111	46	65	0.016	51	11	40	0.36	30	16	14	0.12
Gleason grading	8≤	59	36	23		20	7	13		9	8	1	
Post-operative	7≥	88	31	57	<0.001	55	12	43	0.21	32	18	14	0.22
Gleason grading	8≤	82	51	31		16	6	10		7	6	1	
Biopsy positive core rate	Mean (SD)	35.8 ± 23.1	40.6 ± 24.5	31.9 ± 21.3	0.025	30.6 ± 20.7	36.1 ± 28.0	28.8 ± 17.6	0.31	27.6 ± 18.4	33.7 ± 19.8	17.9 ± 10.3	0.002
IDC-P (biopsy)	Positive	22	18	4	<0.001	7	4	3	0.064	9	8	1	0.12
	Negative	148	64	84		64	14	50		30	16	14	
Cribriform pattern (biopsy)	Positive	37	26	11	0.003	6	4	2	0.033	5	4	1	0.63
	Negative	133	56	77		65	14	51		34	20	14	

*SD* standard deviation, *PSA* prostate-specific antigen, *TPV* total prostate volume, *BCR* biochemical recurrence, *IDC-P* intraductal carcinoma of the prostate, *NMSH* Nippon Medical School Hospital, *AMUH* Aichi Medical University Hospital, *JUH* Juntendo University Hospital.

### Key feature generation and pathological characterization

**Fig. 1:**
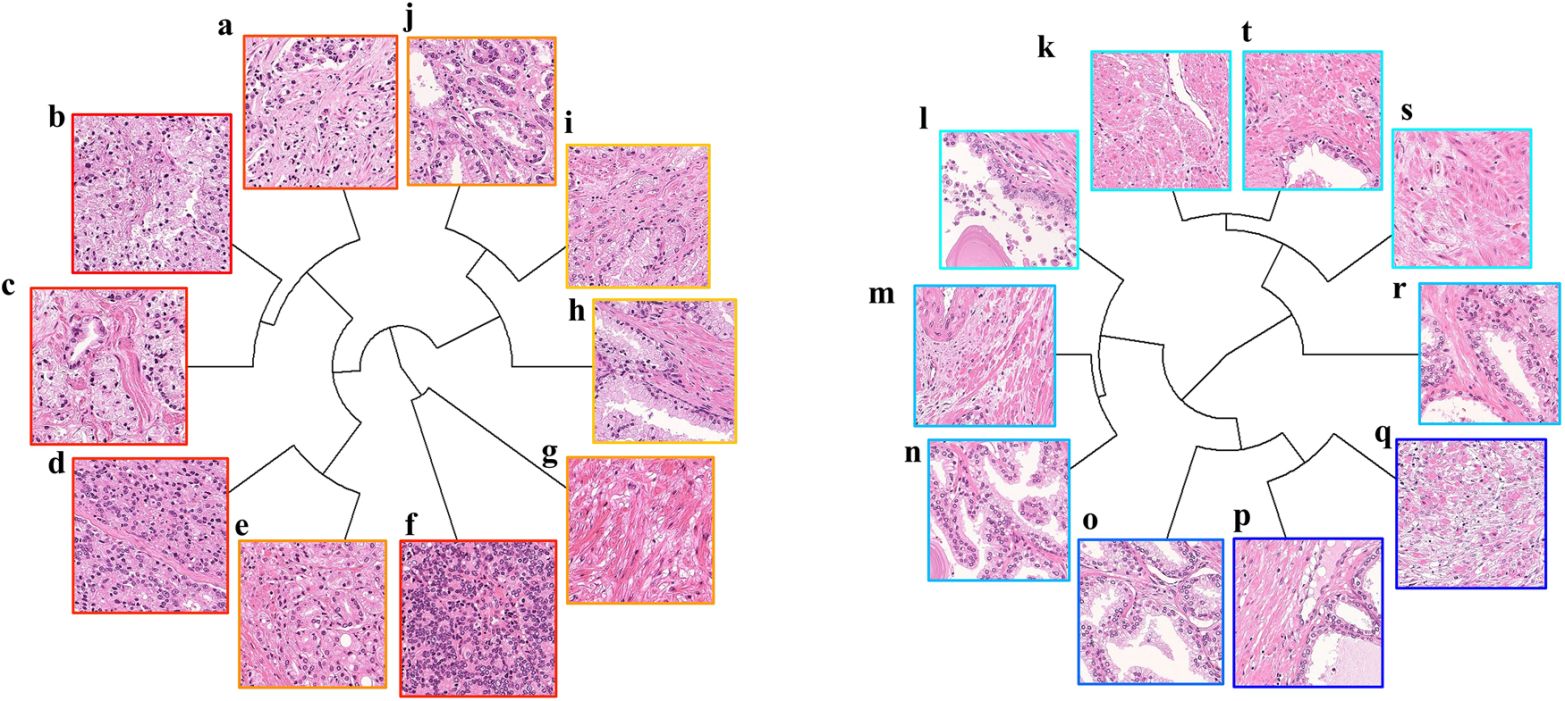
Representative images of key features. Top ten images with the highest and lowest ranking scores. **a–j** Represent the BCR group and **k–t** represent the non-BCR group. Most features in the BCR group contained high-grade Gleason pattern components and benign stromal and smooth muscle components. No cancerous components were detected in the non-BCR group. **a**, **b**, **d**, **f** Cancers consisting of Gleason pattern 4 or 5, indicating aggressive clinical behavior. **c**, **e** Cancers consisting predominantly of Gleason pattern 4, which indicates an unfavorable clinical behavior. **i**, **j** Cancers with the predominant Gleason pattern 3. **g**, **h**, **k**–**s**, and **t** Acini and stromal components without cancer cells. BCR biochemical recurrence.

We generated 100 key features through a single-step analysis of intermediate magnification images, contrary to our previous study that employed a two-step process at low and high magnifications^10^. The two studies differ in the use of Vision Transformer (ViT), with our current study in comparison to an autoencoder. The top ten representative pathological images with the highest and lowest impact scores are shown in Fig. 1. Positive findings were characterized by the presence of high-grade Gleason components. Stromal and smooth muscle elements were also observed without cancer cells. Furthermore, none of the bottom ten images contained cancerous cells.

### Evaluation of generalizability and clinical utility

**Fig. 2:**
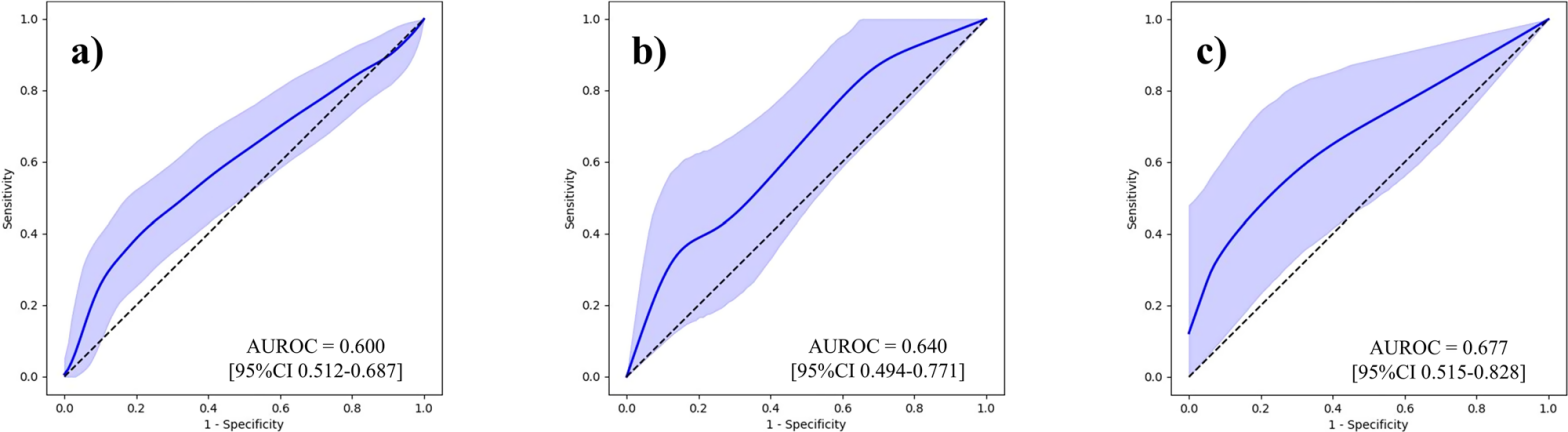
ROC curves for the BCR prediction using only the Gleason grading. **a** NMSH, **b** AMUH, **c** JUH The blue line represents the ROC (bootstrapped) curves for the BCR prediction using only the Gleason grading for each institution. The blue shaded region indicates the 95% CI for the ROC curve. The AUROCs and 95% CI were estimated using 10,000 bootstrap resamples. ROC receiver operating characteristic, BCR biochemical recurrence, AUROC area under the receiver operating characteristic curve, CI confidence interval. NMSH Nippon Medical School Hospital, AMUH Aichi Medical University Hospital, JUH Juntendo University Hospital.

**Fig. 3:**
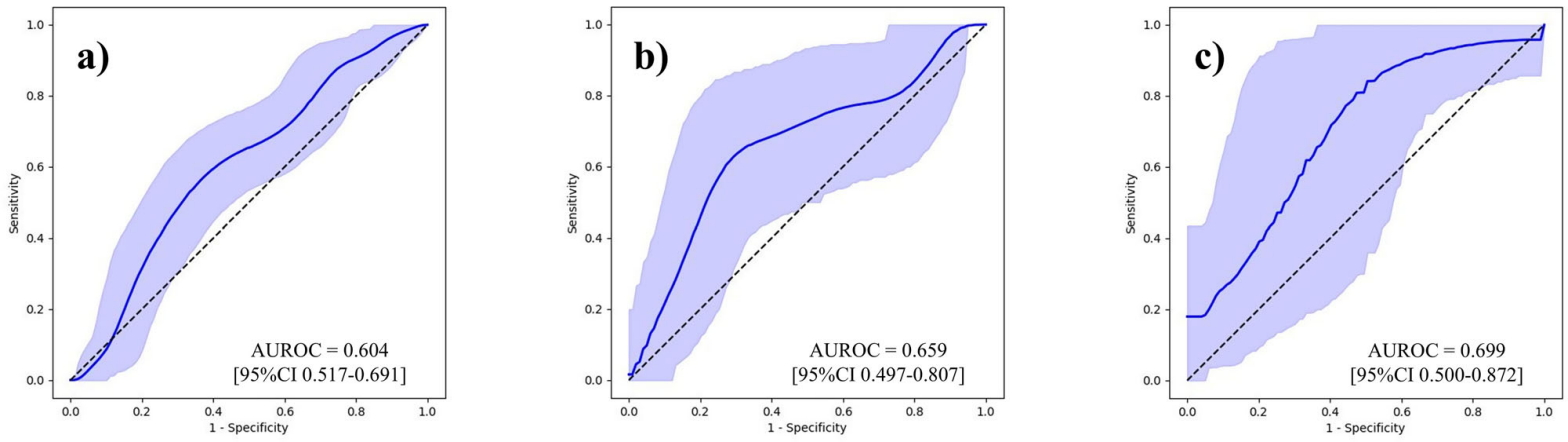
ROC curves for the BCR prediction using tabular data of 100 variables directly. **a** NMSH, **b** AMUH, **c** JUH. The blue line represents the ROC (bootstrapped) curves for the BCR prediction using tabular data of 100 variables directly for each institution. The blue shaded region indicates the 95% CI for the ROC curve. The AUROCs and 95% CI were estimated using 10,000 bootstrap resamples. ROC receiver operating characteristic, BCR biochemical recurrence, AUROC area under the receiver operating characteristic curve, CI confidence interval. NMSH Nippon Medical School Hospital, AMUH Aichi Medical University Hospital, JUH Juntendo University Hospital.

**Fig. 4:**
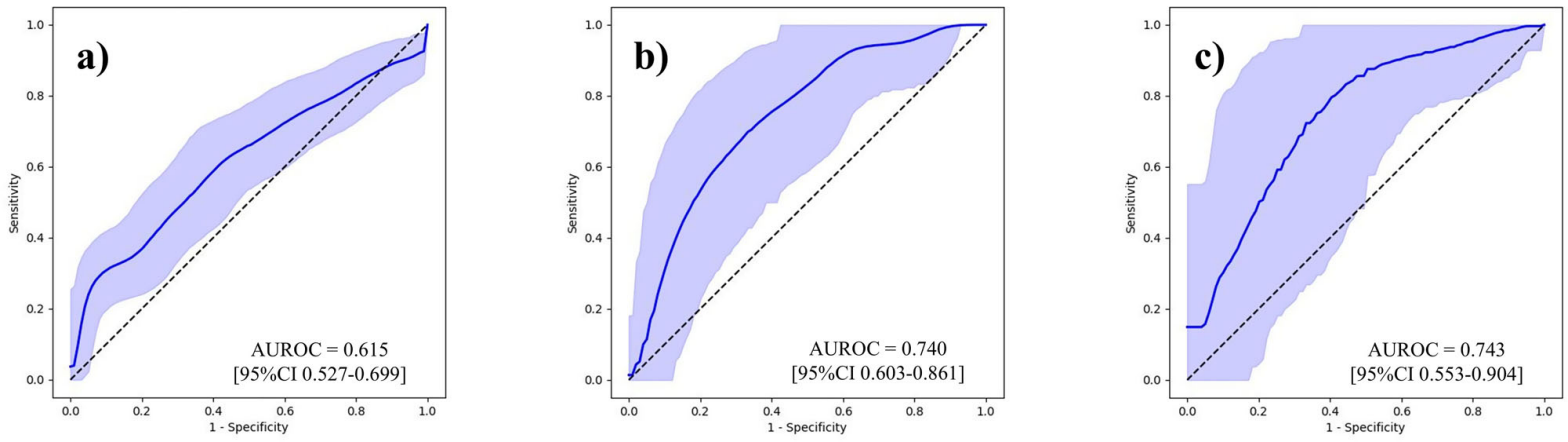
ROC curves for the BCR prediction using the ML-predicted Gleason grading. **a** NMSH, **b** AMUH, **c** JUH. The blue line represents the ROC (bootstrapped) curves for the BCR prediction using ML-predicted Gleason grading for each institution. The blue shaded region indicates the 95% CI for the ROC curve. The AUROCs and 95% CI were estimated using 10,000 bootstrap resamples. ROC receiver operating characteristic, BCR biochemical recurrence, ML machine learning, AUROC area under the receiver operating characteristic curve, CI confidence interval. NMSH Nippon Medical School Hospital, AMUH Aichi Medical University Hospital, JUH Juntendo University Hospital.

**Fig. 5:**
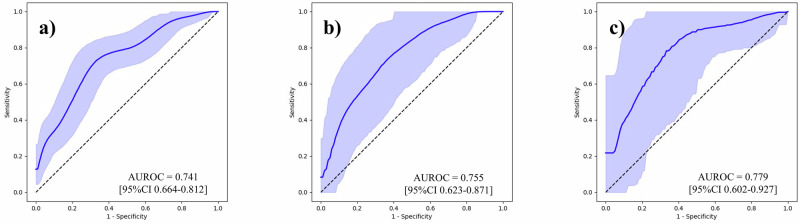
ROC curves for the BCR prediction using the ML-predicted reasoning-oriented score. **a** NMSH, **b** AMUH, **c** JUH. The blue line represents the ROC (bootstrapped) curves for the BCR prediction using the ML-predicted reasoning-oriented score for each institution. The blue shaded region indicates the 95% CI for the ROC curve. The AUROCs and 95% CI were estimated using 10,000 bootstrap resamples. ROC receiver operating characteristic, BCR biochemical recurrence, ML machine learning, AUROC area under the receiver operating characteristic curve, CI confidence interval. NMSH Nippon Medical School Hospital, AMUH Aichi Medical University Hospital, JUH: Juntendo University Hospital.

**Fig. 6:**
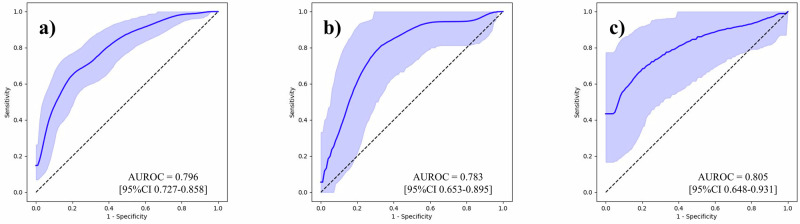
ROC curves for the BCR prediction using the combination of PSA and ML-predicted reasoning-oriented score. **a** NMSH, **b** AMUH, **c** JUH. The blue line represents the ROC (bootstrapped) curves for the BCR prediction using the combination of PSA and ML-predicted reasoning-oriented scores for each institution. The blue shaded region indicates the 95% CI for the ROC curve. The AUROCs and 95% CI were estimated using 10,000 bootstrap resamples. ROC receiver operating characteristic, BCR biochemical recurrence, ML machine learning, AUROC area under the receiver operating characteristic curve, CI confidence interval. NMSH Nippon Medical School Hospital, AMUH Aichi Medical University Hospital, JUH Juntendo University Hospital.

To underscore the effect of our feature generation process, we first conducted BCR predictions using the Gleason grading assigned to each sample, and then using tabular data that contained 100 variables per sample. Supplementary Table 1 outlines the follow-up periods for each institution. Area under the receiver operating characteristic curves (AUROC) with its 95% confidence interval (CI) was used as the evaluation metric. The results are outlined in Table 2, and the ROC curves are shown in Figs. 2–6. The AUROCs (bootstrapped) for BCR prediction using only Gleason grading assigned to each sample were 0.600 (95% CI 0.512–0.687; bootstrap *n* = 10,000), 0.640 (95% CI 0.494–0.771; bootstrap *n* = 10,000), and 0.677 (95% CI 0.515–0.828; bootstrap *n* = 10,000) for the NMSH, AMUH, and JUH cohorts, respectively. Extracting tabular data on the positivity of each feature per sample and inputting them for the prediction of BCR yielded AUROCs of 0.604 (95% CI 0.517–0.691; bootstrap *n* = 10,000), 0.659 (95% CI 0.497–0.807; bootstrap *n* = 10,000), and 0.699 (95% CI 0.500–0.872; bootstrap *n* = 10,000) for the NMSH, AMUH, and JUH cohorts, respectively. We further investigated whether utilizing the intermediate reasoning step, whereby we predicted the Gleason grading or reasoning-oriented score, and inputting these scores into a linear regression could improve BCR prediction. Predicting the Gleason grading from the tabular distribution of 100 features per sample using this resultant score for BCR prediction yielded AUROCs of 0.615 (95% CI 0.527–0.699; bootstrap *n* = 10,000), 0.740 (95% CI 0.603–0.861; bootstrap *n* = 10,000), and 0.743 (95% CI 0.553–0.904; bootstrap *n* = 10,000) for the NMSH, AMUH, and JUH cohorts, respectively. Using the reasoning-oriented score as an intermediate step increased each model’s predictive capacity, with AUROCs of 0.741 (95% CI 0.664–0.812; bootstrap *n* = 10,000), 0.755 (95% CI 0.623-0.871; bootstrap *n* = 10,000), and 0.779 (95% CI 0.602–0.927; bootstrap *n* = 10,000) for the NMSH, AMUH, and JUH cohorts, respectively. Finally, integrating PSA values led to the highest AUROCs for all models: 0.796 (95% CI 0.727–0.858; bootstrap *n* = 10,000), 0.783 (95% CI 0.653-0.895; bootstrap *n* = 10,000), and 0.805 (95% CI 0.648–0.931; bootstrap *n* = 10,000) for the NMSH, AMUH, and JUH cohorts, respectively. Next, we calculated the Brier score as a measure of overall calibration (Table 3). Across all cohorts, the ML-predicted reasoning-oriented score consistently reduced the Brier score compared with directly inputting 100 variables (NMSH: from 0.360 to 0.219; AMUH: from 0.295 to 0.173; JUH: from 0.480 to 0.239). Furthermore, the best performance was achieved when combining PSA with the ML-predicted reasoning-oriented score, with the Brier score decreasing from 0.242 (Gleason only) to 0.194 in the NMSH cohort, from 0.179 to 0.158 in the AMUH cohort, and from 0.219 to 0.214 in the JUH cohort. These findings demonstrate that the intermediate reasoning step not only improved discriminative ability but also enhanced the calibration of predicted probabilities across institutions. In addition, calibration plots showed that the model combining the ML-predicted reasoning-oriented score with PSA tracked the 45-degree reference line more closely than comparator models across NMSH, AMUH, and JUH (Supplementary Fig. 1). Furthermore, decision-curve analysis demonstrated that this combined model provided a consistently higher net benefit than the comparator models across institutes and over a clinically relevant range of threshold probabilities (Supplementary Fig. 2). Supplementary Table 2 summarizes the results of multivariable logistic regression with pre-operative covariates. Among NMSH and AMUH, the ML-predicted reasoning-oriented score remained an independent predictor of BCR with the highest odds ratio among significant predictors per each cohort (*p* = 0.0001 for NMSH and *p* = 0.0143 for AMUH). As shown in Supplementary Fig. 3, the AUROC values for BCR predictions using the Kattan nomogram were 0.752 (95% CI, 0.678–0.823; bootstrap *n* = 10,000), 0.639 (95% CI, 0.457–0.808; bootstrap *n* = 10,000), and 0.755 (95% CI, 0.588–0.898; bootstrap *n* = 10,000) for NMSH, AMUH, and JUH, respectively. Our ML-based model achieved superior performance in all three cohorts.

**Table 2:** AUROCs for BCR predictions

BCR prediction	Gleason grading only	Tabular data of 100 variables directly	ML-predicted Gleason grading	ML-predicted reasoning-oriented score	Combination of PSA and ML-predicted reasoning-oriented score
NMSH	0.600 [95% CI 0.512–0.687]	0.604 [95% CI 0.517–0.691]	0.615 [95% CI 0.527–0.699]	0.741 [95% CI 0.664–0.812]	0.796 [95% CI 0.727–0.858]
AMUH	0.640 [95% CI 0.494–0.771]	0.659 [95% CI 0.497–0.807]	0.740 [95% CI 0.603–0.861]	0.755 [95% CI 0.623–0.871]	0.783 [95% CI 0.653–0.895]
JUH	0.677 [95% CI 0.515–0.828]	0.699 [95% CI 0.500–0.872]	0.743 [95% CI 0.553–0.904]	0.779 [95% CI 0.602–0.927]	0.805 [95% CI 0.648–0.931]

The AUROCs and 95% CIs were estimated using 10,000 bootstrap resamples. *AUROC* area under the receiver operating characteristic curve, *BCR* biochemical recurrence, *ML* machine learning, *PSA* prostate-specific antigen, *CI* confidence interval. *NMSH* Nippon Medical School Hospital, *AMUH* Aichi Medical University Hospital, *JUH* Juntendo University Hospital.

**Table 3:** Brier scores for predictive models

BCR prediction	Gleason grading only	Tabular data of 100 variables directly	ML-predicted Gleason grading	ML-predicted reasoning-oriented score	Combination of PSA and ML-predicted reasoning-oriented score
NMSH	0.242	0.360	0.242	0.219	0.194
AMUH	0.179	0.295	0.185	0.173	0.158
JUH	0.219	0.480	0.327	0.239	0.214

*BCR* biochemical recurrence, *ML* machine learning, *PSA* prostate-specific antigen. *NMSH* Nippon Medical School Hospital, *AMUH* Aichi Medical University Hospital, *JUH* Juntendo University Hospital.

## Discussion

In this study, we implemented an intermediate reasoning step that enhanced the prediction of clinical outcomes of prostate cancer. The proposed approach comprises three key steps. First, we used a ViT-based model to extract histopathological features from prostate tissue slides. Second, we leveraged these features to generate a reasoning-oriented score to guide the direction of intermediate reasoning, which combines information from histopathological features with established prognostic knowledge. Third, we used this score to predict BCR and demonstrated its robust and generalizable performance across external validation datasets. The effectiveness of this pipeline confirmed that embedding intermediate reasoning steps enhanced the performance of ML-based histopathological image prognostication. Importantly, we validated the generalizability of our protocol using multiregional external validation datasets, highlighting its potential to provide equitable prognostication. Furthermore, while our feature extraction algorithm was based on a dataset of prostate whole-mount sections, the prognostication component required only a limited biopsy sample cohort for training.

The reasoning-oriented score differs subtly from Gleason grading; however, this small, structured modification enabled by intermediate reasoning can result in a meaningful, robust, and more equitable improvement in prognostic performance by capturing latent histopathological signals. The rationale for implementing an intermediate step in this study was multifold. The feature space was substantially reduced to a single number instead of 100 variables. Inputting an abundance of features into a model oftentimes yields high performance; however, this comes with the risk of overfitting to the training dataset and being inept at domain-shift tasks^16^. Our proposed system circumvents this by consolidating all learned features into a single score built upon a clinically established prognostic classification system. A direct supervised learning approach using Gleason grading as the label may appear to be a straightforward strategy; however, there are several challenges to this approach. First, the Gleason grading system is inherently discrete, whereas the histopathological features manifest along a continuous spectrum. If the model is trained directly on discrete Gleason grading, it may struggle to learn subtle transitions between patterns, potentially reducing its ability to be generalized across diverse cases. Second, inter-observer variability is a well-documented issue in Gleason grading. Inter-observer variability among pathologists in assigning scores to the same specimen may introduce label noise, thereby compromising the robustness of the model. Direct training on such subjective labels may lead to the overfitting of individual grading tendencies rather than learning generalizable pathological features. Third, the Gleason grade alone is an imperfect predictor of clinical outcomes. Although Gleason grade serves as a strong prognostic marker, patients with the same Gleason grade may exhibit significantly different recurrence risks. By allowing the ML model to extract histopathological features independently and perform intermediate reasoning using a reasoning-oriented score, we aimed to mitigate these challenges and enhance the predictive performance. We believe that this process enhanced the model’s generalizability and validated it through trials on multiregional external datasets from AMUH and JUH, comprising biopsy samples rather than whole-mount sections. As ML tools are increasingly integrated into clinical practice, digital health equity remains a critical concern. Reliance on homogeneous training datasets and limited access to such technologies hinder equitable implementation, particularly in resource-constrained regions. Our pipeline, designed to be both interpretable and generalizable to diverse external datasets, supports the broader and more equitable adoption of ML in global healthcare settings. Our approach addresses domain shifts in a clinically meaningful and technical manner. We consider this medically grounded mitigation of domain shifts a core contribution to fairness in medical informatics. This study represents a step toward bridging the disparities in access to high-performance diagnostic tools. From a research perspective, our pipeline enables development in data-limited environments without extensive manual annotations. From a clinical perspective, this addresses dual domain shifts—across specimen types (biopsy vs. prostatectomy) and across institutions—thereby providing standardized predictions that reduce inter-observer variability and ensure applicability across diverse clinical settings. Balancing interpretability and training efficiency offers a scalable solution for real-world clinical implementations, particularly in resource-limited settings.

We incorporated several design elements into our pipeline to realize an equitable and generalizable prognostication. One such strategy involves leveraging knowledge acquired from whole-mount sections and utilizing it for the prediction of clinical outcomes based only on biopsy samples. This has conventionally been difficult because of the differences between these two types of histopathological samples, including their preparation and appearance. For instance, cell nuclei could have varying appearances between whole-mount sections and biopsy samples owing to differences in formaldehyde fixation. There is a substantial benefit to this approach, as clinicians may apply knowledge acquired from post-surgical whole-mount sections and apply it to analyze pre-surgical biopsy samples. While biopsy samples primarily consist of the target tumor tissue, whole-mount sections contain a greater volume of pathological information, even those distant from the tumor tissue, including the interstitial tissue. Utilizing such data leads to the acquisition of additional data that is not attainable with the use of biopsy samples only, thus allowing for a potential improvement in prognostic capacity.

Another central design element of our pipeline is its unique approach to generating 100 histopathological features. We previously established an unsupervised framework using deep autoencoders for feature extraction. This study explored a supervised alternative using BCR labels and ViT^10,17^. Although both approaches reduce the feature space to 100 dimensions, they provide complementary perspectives on feature generation and interpretation. In this study, we utilized the BCR as a training label for the feature extraction step. However, in our previous study, we did not use any labeled data for this step because we employed a deep autoencoder trained in an unsupervised manner^10^. Such changes represent the difference between supervised and unsupervised learning, utilized in our feature extraction step that occurs before the intermediate and final BCR prognostication steps. Each method has its advantages and disadvantages. In supervised learning, an algorithm is trained to classify samples according to labels provided. In this study, the features are influenced by the BCR labels and thus can increase the prognostication performance in subsequent steps; however, this feature generation process is bound by information on the BCR. In unsupervised learning, we overcame this limitation by allowing the model to discover a means of clustering different features on its own without target labels. This may ultimately yield a lower classification or predictive capacity, although it can lead to the discovery of new features that are not associated with the labels. These differences in methodology between the current and previous studies yielded changes in the composition of the histopathological features. Each approach has its advantages and should be selected appropriately for the task at hand.

This study had some limitations. First, although our cohort was limited to Japanese patients, who are typically underrepresented in large ML projects such as the PANDA challenge, their inclusion offers a valuable perspective toward equitable and inclusive ML development. However, ongoing investigations using multiethnic cohorts are necessary to ensure broader applicability. Second, although our pipeline incorporates histopathological and PSA information, additional integration with imaging, genomic, or broader clinical variables could further enhance the predictive performance. In the future, we plan to conduct more extensive investigations to confirm the applicability of our protocol to a larger sample set of diverse patients to fully assess its clinical utility.

We demonstrated that incorporating an intermediate reasoning step can enhance the performance and generalizability of ML-based histopathological image analysis, even with limited training data. Our approach uniquely combines ML with established clinical knowledge in the form of a reasoning-oriented score, resulting in an improved prognostic accuracy for clinical outcomes. The robust performance of the model across external validation datasets addresses the key challenges in medical ML: the scarcity of large-scale medical datasets and the need for generalizability across institutions. Furthermore, by maintaining interpretability while achieving superior predictive performance, our method bridges the gap between advanced ML techniques and clinical practice. This framework has potential applications across various disease domains beyond prostate cancer, offering a promising direction for developing more efficient and equitable ML-based diagnostic tools while leveraging valuable insights accumulated through decades of clinical experience.

## Methods

### Study population

A total of 380 patients who underwent RP for localized prostate cancer at NMSH, AMUH, and JUH were enrolled, with 270, 71, and 39 patients included from each institution, respectively. Patients from the NMSH were enrolled between 2000 and 2016, those from the AMUH between 2015 and 2018, and those from the JUH between 2012 and 2021. None of the patients were enrolled in clinical trials involving RP or received neoadjuvant or adjuvant therapy. Patients who could not be followed up within one year because of hospital transfer or death owing to other causes were also excluded. Among the NMSH cohorts, the pathological features were extracted from 100 patients using the novel algorithm. Among these patients, we selected prostate whole-mount sections with the largest specimen area per patient, yielding 100 whole-mount sections for analysis. Following this, we predicted BCR using biopsy specimens from the remaining 170 NMSH cases, along with AMUH and JUH cases. Whole-mount prostatectomy sections were used exclusively for supervised feature extraction to obtain key features and were not used for training or testing the BCR prediction models.

### Statistical analysis

The Wilcoxon rank-sum test was used for continuous data. Categorical variables were analyzed using Fisher’s exact test. We performed multivariable logistic regression using pre-operative clinical factors (age, clinical T stage, and PSA) and the ML-predicted reasoning-oriented score. The multivariable logistic regression was conducted using JMP version 14.2.0 (SAS Institute Inc., Cary, NC, USA), and two-sided *p*-values < 0.05 were considered statistically significant.

### Acquisition of histopathological samples

Prostate biopsy specimens were obtained according to standard biopsy protocols of each institution. Systematic and targeted biopsies were performed under transrectal ultrasound guidance using needle biopsy devices to ensure accurate prostate tissue collection. Prostatectomy specimens were obtained according to established institutional protocols for RP. These specimens were promptly processed for histopathological examination, including Gleason grading of the prostate.

### Preparation of pathology images

The prostate tissues were fixed in 10% formalin and embedded in paraffin. Samples were then sectioned at a thickness of 3 μm and underwent hematoxylin and eosin (H&E) staining. All slides were scanned using a whole-slide imaging scanner (Hamamatsu NanoZoomer S60 Slide Scanner) equipped with a x20 objective lens and stored securely on a dedicated computer. Histopathological images were analyzed at intermediate magnification (×50).

### Histological grading

We classified prostate cancer based on the International Society of Urological Pathology (ISUP) classification criteria^18^. Pathologists assessed each sample based on both the Gleason score and Gleason grade groups. In the internal validation cohort, the Gleason score yielded superior predictive capacity; thus, the Gleason score was used as the Gleason grading system in this study. Samples from NMSH, AMUH, and JUH were independently evaluated by pathologists at each institution.

### Definition of biochemical recurrence

Post-RP BCR was defined based on the guidelines from the European Association of Urology^19^. All patients were followed up at their first BCR evaluation within six weeks post-RP and subsequently evaluated every three months. Positive BCR was defined as post-RP PSA levels above 0.2 ng/mL on two consecutive evaluations. If PSA values were above 0.2 ng/mL at the first post-RP visit, the date of surgery was recorded as the date of BCR.

### Overview of our prediction pipeline

**Fig. 7:**
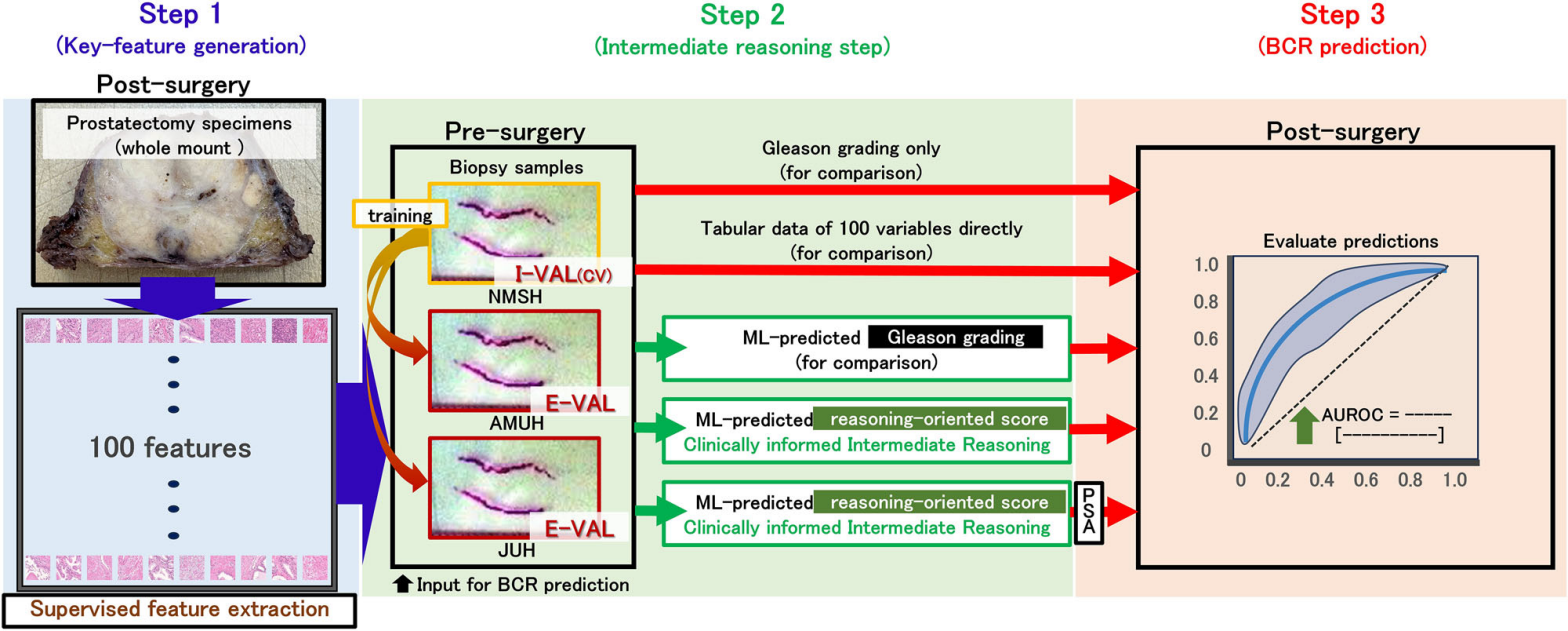
Overview of the analytical pipeline. Step 1: Key feature generation; Step 2: Intermediate reasoning; Step 3: BCR prediction. ML machine learning, CV cross-validation, I-VAL internal validation, E-VAL external validation, PSA prostate-specific antigen, BCR biochemical recurrence, AUROC area under the receiver operating characteristic curve. NMSH Nippon Medical School Hospital, AMUH Aichi Medical University Hospital, JUH Juntendo University Hospital.

**Fig. 8:**
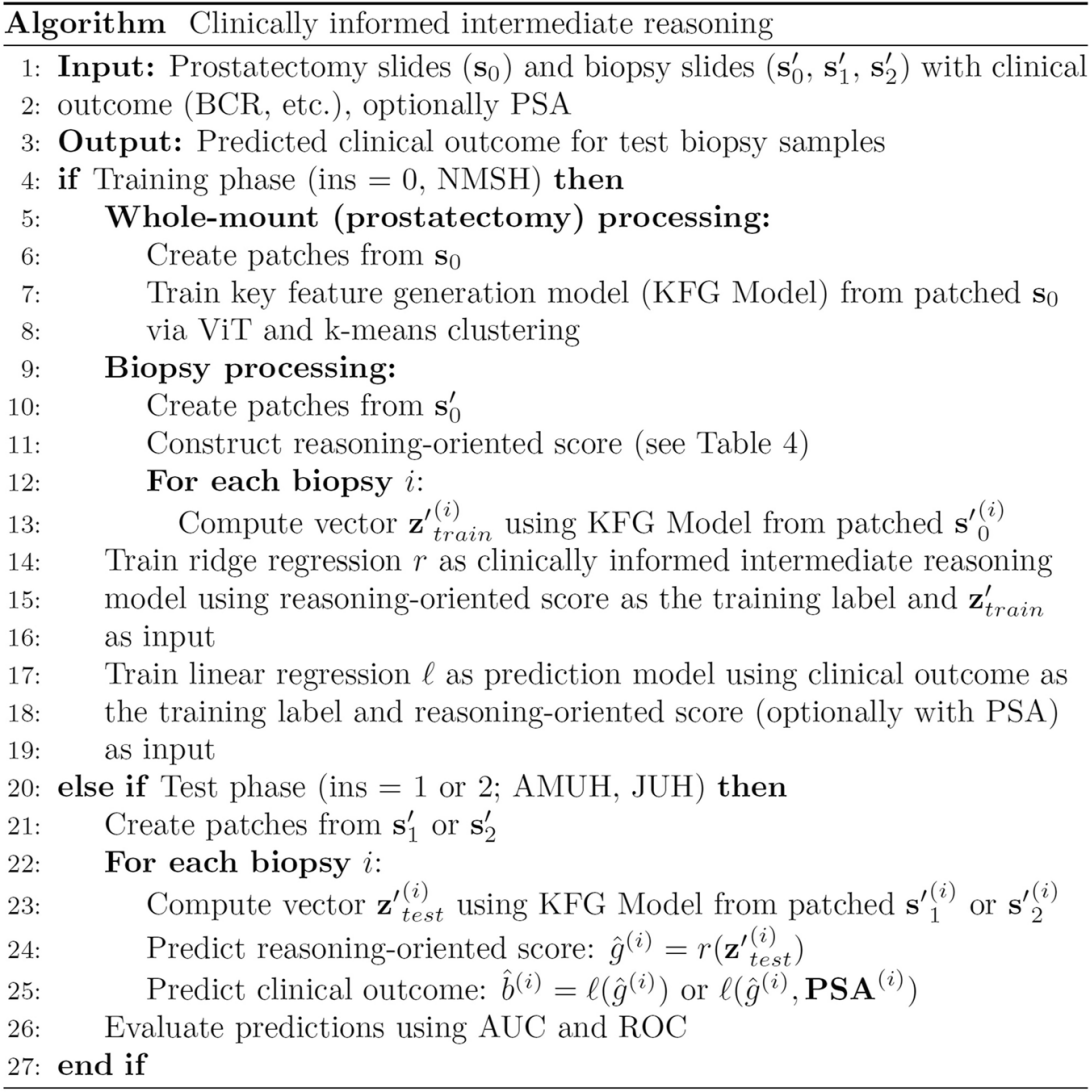
Pseudocode of our clinically informed intermediate reasoning. This algorithm illustrates the processes of key feature generation, intermediate reasoning through a reasoning-oriented score, and clinical outcome prediction. Clinical outcome (BCR, etc.) is used only as a training label and not used during inference.

An overview of our prediction pipeline, ranging from key feature generation and intermediate reasoning via a reasoning-oriented score to BCR prediction, is shown in Fig. 7. Figure 8 shows the detailed pseudocode for our clinically informed intermediate reasoning pipeline. The training and test phases were clearly delineated, and optional integration with PSA values was also considered to enhance the clinical applicability of BCR prediction. The subsequent sections provide further details regarding the individual components.

### Key feature generation

**Fig. 9:**
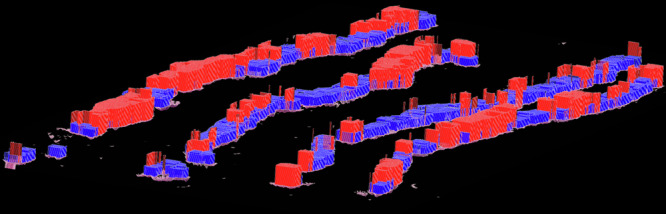
Automatically annotated biopsy sample. Our algorithm automatically generated key features based on whole-mount pathological images without manual annotation. This figure represents a three-dimensional feature map of the biopsy sample. The regions with impact scores above and below 0.5 are shaded in red and blue, respectively, while the height of each bar represents its impact score.

Our novel ML algorithm proposed for this study utilized a ViT for the extraction of key features^17^. As aforementioned, we selected 100 whole-mount sections belonging to 100 patients from NMSH (Supplementary Table 3), which were labeled according to the time to BCR. First, the images were divided into 3,507,928 patches. The patches were designed such that they did not overlap during the training. These were then input into a ViT-based algorithm programmed to conduct a binary classification task to predict whether the samples belonged to positive or negative BCR groups. Importantly, this process was conducted in a supervised manner, contrary to our prior studies that utilized deep autoencoders in an unsupervised manner^10^. We added an extra layer with 2048 nodes to the end of the multilayer perceptron component of the ViT, which then connected to the final binary output layer with designated labels. This layer contained vectors that represent important features for classification tasks and was thus extracted as an input for the following task. All hyperparameters of the ViT are provided in Supplementary Table 4. Subsequently, these vectors were reduced to 100 feature clusters using k-means clustering, yielding 100 clusters, each representing a key feature utilized for the subsequent BCR classification task. Representative images of these key features are shown in Fig. 1. We set the number of clusters to 100 based on preliminary experiments. Although k-means involves stochastic initialization, we confirmed that the downstream performance remained stable across runs. The impact score was calculated as in our previous study^10^. For each feature, a representative image was selected from among the three images that were closest to the centroid of each cluster per pathologist evaluation. The images were ranked according to their impact scores. The top ten images with the highest and lowest ranking scores were selected for display in the figure. The image sizes were adjusted according to the equation |0.5-impact score| + 2.5. The R package APE: Analyses of Phylogenetics and Evolution (version 4.4.0) was used for visualization. Figure 9 displays a three-dimensional feature map of a biopsy sample. The regions with impact scores above and below 0.5 calculated as above, are shaded in red and blue, respectively, while the height of each bar represents its impact score. The feature extraction model was based solely on prostatectomy specimens, while biopsy cohorts were completely independent and reserved for external validation, thereby avoiding information leakage.

### Reasoning-oriented score

We developed a reasoning-oriented score to guide intermediate reasoning. This was accomplished by first assigning different combinations of Gleason scores from six to 10 to a new grading scale. For each sample belonging to the training dataset, the labels were adjusted such that if they had a positive BCR, an extra point was added to the Gleason grading, whereas if they had a negative BCR, one point was subtracted. Hence, the final scale consisted of grades 1–11 (Table 4), which was scaled and predicted in the subsequent steps. Importantly, the ML-predicted reasoning-oriented score does not incorporate future knowledge of BCR status for samples undergoing prognostication. Once defined in the training set, it can be directly applied to new biopsy specimens based on histopathology images, supporting its feasibility in prospective clinical settings.

**Table 4:** Assignment of the new grading scale based on the Gleason grading

Gleason grading (Histologic patterns)	Newly assigned score	Score adjustment
≤6 (3 + 3)	2	
7 (3 + 4)	3	
7 (4 + 3)	4	
8 (4 + 4)	5	
8 (3 + 5)	6	±1 based on BCR
9 (4 + 5)	7	
8 (5 + 3)	8	
9 (5 + 4)	9	
10 (5 + 5)	10	

Each training sample was assigned a new score based on detailed histologic pattern combinations. An extra score was added or subtracted from this newly assigned score based on the BCR information. This yielded a reasoning-oriented score (range 1–11), which was scaled and predicted in the subsequent steps. Importantly, during inference, the reasoning-oriented score was always predicted without access to BCR, so no data leakage occurred. BCR biochemical recurrence.

### Intermediate reasoning step

The key features were used to score each testing sample with a reasoning-oriented score. This analysis was conducted on three different cohorts. The first cohort comprised 170 biopsy images from patients with NMSH after excluding 100 cases used for key feature generation. The other two cohorts comprised 71 AMUH and 39 JUH biopsy images. For this step, we used only biopsy samples, rather than whole-mount sections. The biopsy samples were divided into 33,937,419 (NMSH), 11,618,974 (AMUH), and 6,949,818 (JUH) patches, with each patch designed to overlap. Each patch was assigned to one feature cluster. The number of patches belonging to each cluster was tallied and divided by the total number of patches per sample, generating tabular data with 100 variables, each representing the percentage of patches belonging to each cluster. The total number of patches per sample was calculated after removing patches deemed background and that did not contain histopathological samples. These tabular data were then input into a ridge regression and trained to predict either the Gleason grading as a target for comparison or the reasoning-oriented score. We defined the process of inputting histopathological-feature-derived data into ML and predicting a grading score as an intermediate reasoning step. This step represents a reasoning process whereby high-dimensional features are first distilled into a reasoning-oriented score, which is subsequently used for the final outcome prediction.

### BCR prediction

Finally, the 5-year BCR was predicted using linear regression with the following inputs: (1) Gleason grading only, (2) tabular data of 100 variables directly, (3) ML-predicted Gleason grading, (4) ML-predicted reasoning-oriented score, and (5) a combination of PSA and ML-predicted reasoning-oriented score. We analyzed the NMSH cohort as an internal validation through a 3-fold cross-validation. Following this, we conducted external validation using the AMUH and JUH samples. For the latter process, training was conducted solely on the NMSH samples (biopsy); thus, none of the AMUH and JUH samples were used as input for training. In addition, we evaluated the predictive performance of the established Kattan nomogram for each cohort^20,21^. We used Scikit-learn (version 1.6.1; Python version 3.9.13) to evaluate AUROCs, Brier scores, calibration curves, and decision-curve analysis, with updating applied to adjust for outcome incidence^22^. The average AUROCs and 95% CIs were computed by patient-level bootstrapping with 10,000 iterations.

### Ethics declaration

Our study was conducted as a multicenter research project, and unified ethical approval was obtained from all participating sites under review by the Institutional Review Board (IRB) of the lead institution (Central Ethics Committee of the Nippon Medical School Foundation, reference number: M-2022-081). This study complied with all the relevant ethical regulations and was conducted under the principles of the Declaration of Helsinki. Informed consent was obtained from each patient, and the opportunity to opt out of participation was guaranteed.

## Supplementary information

Supplementary Information

## Data Availability

Our institutional review board approved the data collection process and analyses. Restrictions apply to the availability of anonymized patient data used retrospectively for this project, with institutional permission, and are thus not publicly available. All requests for data collected or curated in-house should be made to the corresponding author and evaluated according to the institutional policies to determine whether the requested data are subject to intellectual property or patient privacy obligations.

## Code availability

The Python code to implement the Clinically-Informed-Intermediate-Reasoning is available: https://github.com/AIP-BSS/Clinically-Informed-Intermediate-Reasoning.git.
