# Supplementary material for: Clinically informed intermediate reasoning enables generalizable prostate cancer prognostication through machine learning in limited settings
Source: NPJ Digit Med. 2025 Dec 3;9:19. doi: 10.1038/s41746-025-02193-x (PMC12780265; doi:10.1038/s41746-025-02193-x)
Supplement: Supplementary file 1 — Supplementary Information [file 41746_2025_2193_MOESM1_ESM.pdf]

Supplementary Information for

Clinically Informed Intermediate Reasoning Enables Generalizable Prostate Cancer Prognostication through Machine Learning in Limited Settings

Jun Akatsuka, Kotaro Tsutsumi, Mami Takadate, Yasushi Numata, Hiromu Morikawa, Atsushi Marugame, Hayato Takeda, Yuki Endo, Yuka Toyama, Takayuki Takahashi, Kaori Ono, Junya Iwazaki, Ryuji Ohashi, Akira Shimizu, Tomoharu Kiyuna, Maki Ogura, Masao Ueki, Takuma Kato, Toshiyuki China, Mikio Sugimoto, Hisamitsu Ide, Naoto Sassa, Naonori Ueda, Shigeo Horie, Toyonori Tsuzuki, Go Kimura, Yukihiro Kondo, and Yoichiro Yamamoto

Supplementary Figure 1

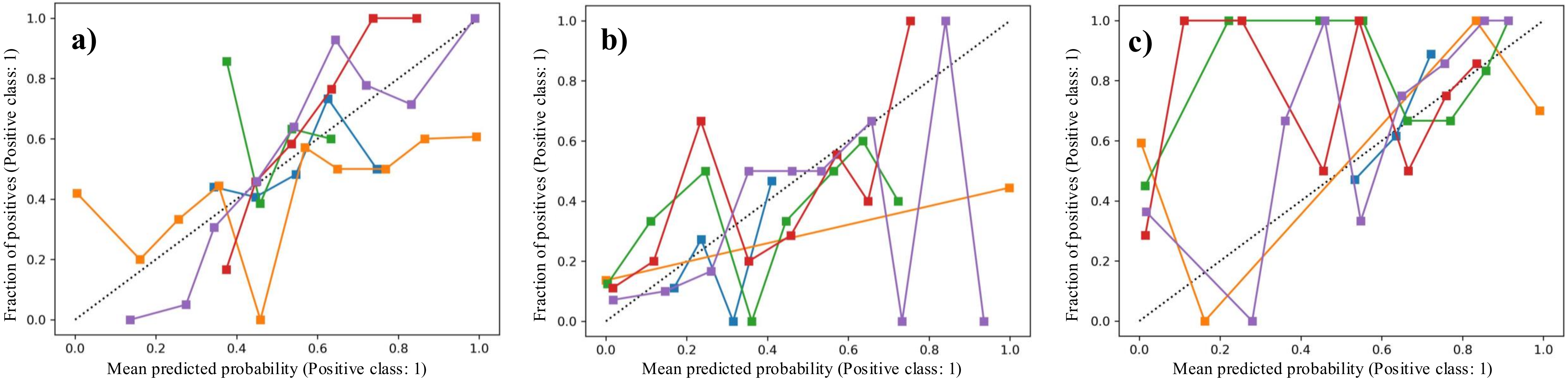

Supplementary Figure 1. Calibration curve for prediction of BCR

a) NMSH, b) AMUH, c) JUH

The gray dotted line represents the perfectly calibrated reference, while the blue, orange, green, red, and purple solid lines correspond to analyses using Gleason grading only, Tabular data of 100 variables directly, ML-predicted Gleason grading, ML-predicted reasoning-oriented score, and combination of PSA and ML-predicted reasoning-oriented score, where each model was updated to adjust for outcome incidence.

BCR: biochemical recurrence, ML: machine learning, PSA: prostate-specific antigen, NMSH: Nippon Medical School Hospital, AMUH: Aichi Medical University Hospital, JUH: Juntendo University Hospital

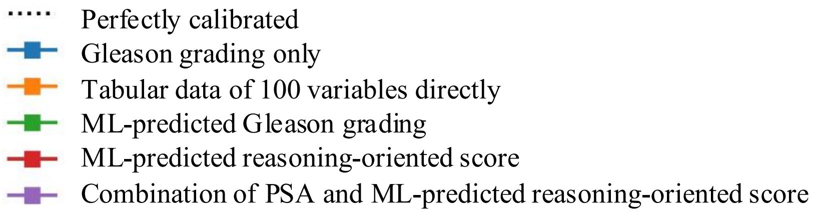

Supplementary Figure 2

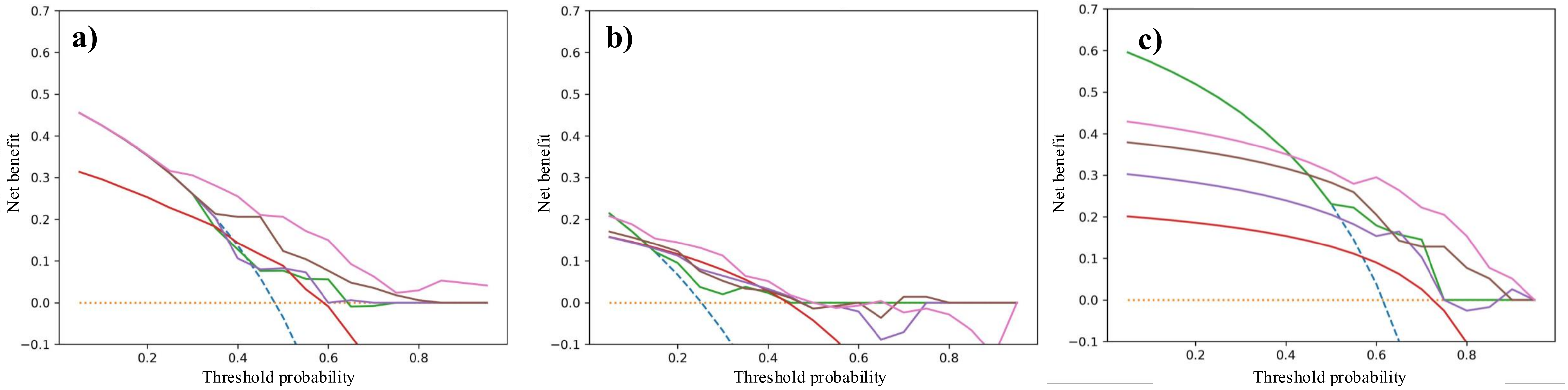

Supplementary Figure 2. Decision curve analysis for prediction of BCR

a) NMSH, b) AMUH, c) JUH

The blue dashed and orange dotted lines represent all positive prediction and all negative prediction strategies, respectively, while the green, red, purple, brown, and pink solid lines correspond to analyses using Gleason grading only, Tabular data of 100 variables directly, ML-predicted Gleason grading, ML-predicted reasoning-oriented score, and combination of PSA and ML-predicted reasoning-oriented score, where each model was updated to adjust for outcome incidence.

BCR: biochemical recurrence, ML: machine learning, PSA: prostate-specific antigen, NMSH: Nippon Medical School Hospital, AMUH: Aichi Medical University Hospital, JUH: Juntendo University Hospital

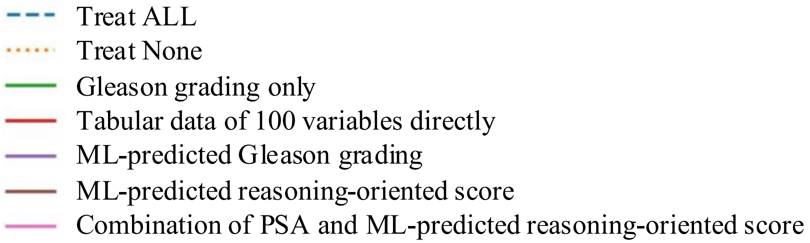

Supplementary Figure 3

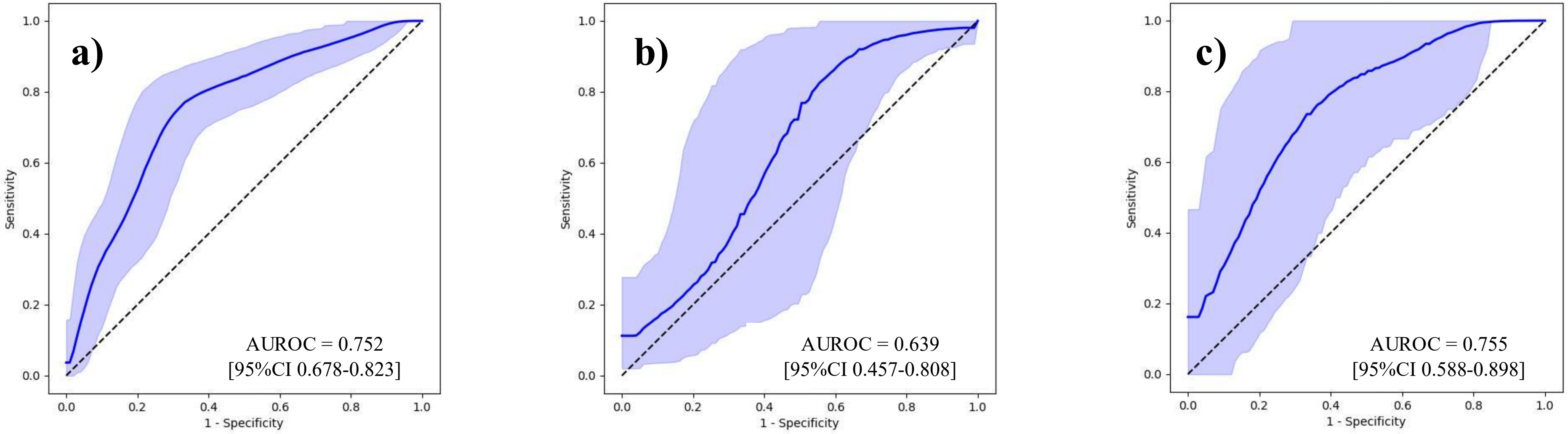

Supplementary Figure 3. ROC curves for the BCR prediction using the Kattan nomogram

a) NMSH, b) AMUH, c) JUH

The blue line represents the ROC curves (bootstrapped) for the BCR prediction using the Kattan nomogram for each institution.

The blue shaded region indicates the 95% CI for the BCR. The AUROCs and 95% CI were estimated using 10,000 bootstrap resamples.

ROC: receiver operating characteristic, BCR: biochemical recurrence, AUROC: area under the receiver operating characteristic curve, CI: confidence interval

NMSH: Nippon Medical School Hospital, AMUH: Aichi Medical University Hospital, JUH: Juntendo University Hospital

Supplementary Table 1 **Follow-up periods for each institution**

		NMSH	AMUH	JUH
Follow-up periods, months	Mean (SD)	96.3 ± 38.2	75.0 ± 14.5	83.0 ± 35.3

*All patients were followed for at least 5 years or until BCR.
BCR: biochemical recurrence, SD: standard deviation, NMSH: Nippon Medical School Hospital, AMUH: Aichi Medical University Hospital, JUH: Juntendo University Hospital

Supplementary Table 2 Multivariable logistic regression analyses for BCR

	NMSH (n=170)		AMUH (n=71)		JUH (n=39)	
	odds ratio 95% CI	p value	odds ratio 95% CI	p value	odds ratio 95% CI	p value
Age	0.94 (0.89-1.00)	0.0509	1.04 (0.93-1.19)	0.506	1.07 (0.88-1.30)	0.501
PSA	1.15 (1.08-1.25)	**0.0001**	1.03 (0.94-1.14)	0.499	1.18 (0.82-1.79)	0.407
Clinical T stage (T2 ≤)	1.49 (0.64-3.48)	0.351	0.67 (0.15-2.87)	0.586	7.94 (0.87-72.21)	0.066
Biopsy positive core rate	1.00 (0.99-1.02)	0.602	1.02 (0.99-1.05)	0.170	1.05 (0.97-1.15)	0.249
IDC-P detected in biopsy	3.63 (0.70-18.92)	0.126	1.61 (0.17-14.99)	0.676	1.02 (0.050-20.56)	0.992
Cribriform pattern detected in biopsy	0.48 (0.13-1.74)	0.267	4.74 (0.45-50.42)	0.197	1.73 (0.049-60.65)	0.762
ML-predicted reasoning-oriented score	2.75 (1.68-4.76)	**0.0001**	2.51 (1.25-5.62)	**0.0143**	1.70 (0.62-5.10)	0.307

BCR: biochemical recurrence, CI: confidence interval, PSA: prostatic specific antigen, IDC-P: intraductal carcinoma of the prostate, ML: machine learning, NMSH: Nippon Medical School Hospital, AMUH: Aichi Medical University Hospital, JUH: Juntendo University Hospital

Supplementary Table 3 Clinical characteristics of the cohort used for pathological feature extraction

Cases, n		n=100
Age, Year	Mean (SD)	66.6 ± 5.63
PSA, ng/ml	Mean (SD)	15.7 ± 19.1
TPV, cm^3	Mean (SD)	32.1 ± 17.2
PSA density, ng/mL/cm^3	Mean (SD)	0.517 ± 0.526
Clinical T stage	T1<br>T2 ≤	24<br>76
Pathological T stage	T2 ≥<br>T3 ≤	60<br>40
Pathological N stage	N0<br>N1	100<br>0
Pre-operative Gleason grading	7 ≥<br>8 ≤	70<br>30
Post-operative Gleason grading	7 ≥<br>8 ≤	61<br>39

SD: standard deviation, PSA: prostate-specific antigen, TPV: total prostate volume

Supplementary Table 4 Hyper parameters of the vision transformer for pathological feature extraction

Network parameters

ViT type: B16

patch_size: 16, hidden size: 768, dropout rate: 0.1, MLP dimension: 3072, num heads: 12, num layers: 12, activation: sigmoid,

normalization: layer normalization, normalization options: epsilon=1e-6,

random normal initalizer stdev in position embedding: 0.06

Solver parameters

optimizer: SGD, learning rate=1e-4, augmentation: none, class-balancing strategy: none, number of epochs: 20
